# Characterization of Immune Infiltration and Construction of a Prediction Model for Overall Survival in Melanoma Patients

**DOI:** 10.3389/fonc.2021.639059

**Published:** 2021-04-02

**Authors:** Gang Li, Xuran Zhu, Chao Liu

**Affiliations:** Plastic Surgery, The Second Affiliated Hospital of Zhengzhou University, Zhengzhou, China

**Keywords:** melanoma, immune infiltration, immunotherapy, tumor microenvironment, prediction model

## Abstract

Reports indicate that the use of anti-programmed cell death-1 (PD-1) and death ligand-1 (PD-L1) monoclonal antibodies for the treatment of patients diagnosed with melanoma has demonstrated promising efficacy. Nonetheless, this therapy is limited by the resistance induced by the tumor microenvironment (TME). As such, understanding the complexity of the TME is vital in enhancing the efficiency of immunotherapy. This study used four different methods to estimate the infiltrating level of immune cells. Besides, we analyzed their infiltration pattern in primary and metastatic melanoma obtained from The Cancer Genome Atlas (TCGA) database. As a consequence, we discovered a significantly higher infiltration of immune cells in metastatic melanoma compared to primary tumor. Consensus clustering identified four clusters in melanoma with different immune infiltration and clusters with higher immune infiltration demonstrated a better overall survival. To elucidate the underlying mechanisms of immune cell infiltration, the four clusters were subdivided into two subtypes denoted as hot and cold tumors based on immune infiltration and predicted immune response. Enrichment analysis of differentially expressed genes (DEGs) revealed different transcriptome alterations in two types of tumors. Additionally, we found tyrosinase-related protein1 (TYRP1) was negatively correlated with CD8A expression. *In vitro* experiments showed that knockdown TYRP1 promoted the expression of HLA-A, B, and C. Eventually, we constructed a prediction model which was validated in our external cohort. Notably, this model also performed effectively in predicting the survival of patients under immunotherapy. In summary, this work provides a deeper understanding of the state of immune infiltration in melanoma and a prediction model that might guide the clinical treatment of patients with melanoma.

## Introduction

Malignant melanoma is one of the most prevalent cancers accounting for up to 1.5% of all cancer-related deaths (1). Melanoma arises from melanocytes, which are found on the skin and mucosal membranes (2). Based on primary tumor location, melanoma is broadly subdivided into cutaneous and non-cutaneous tumors. The former is a rare subtype with an extremely poor prognosis attributed to delayed diagnosis and the aggressive nature of these tumors (3–5). The treatment options for melanoma ranges from surgical excision with free margins, to radiotherapy and chemotherapy (6, 7). In recent years, strides made in the genomic, transcriptomic, and immunological structure of melanoma has enabled the development of novel therapies, thereby causing changes in the paradigm of therapeutic interventions (8, 9).

The current approaches for cancer immunotherapies, led by immune checkpoint inhibitors (ICIs), have shown significant efficacy in patients diagnosed with various cancers (10–12). Malignant melanoma is one of the most immunogenic tumors because of high genomic mutational load, which is considered a benefit from immunotherapy (13–15). An increase of high tumor mutation load produces immunogenic neoantigens, which stimulate immune response (16, 17). Notably, the first immune checkpoint inhibitors, anti-T-lymphocyte-associated protein 4 (Ipilimumab), and programmed cell death legend-1 (Nivolumab) were approved by the US Food and Drug Administration (FDA) in 2011 and 2014, respectively, for the treatment of unresectable or metastatic melanoma (18, 19). Data from clinical trials revealed that the use of immune checkpoint inhibitors prolonged the survival of the patient. A 3-year overall survival rate of patients treated with anti-PD-1 alone or in combination with ipilimumab and a 4-year survival rate for nivolumab plus ipilimumab exceeded 50%. Of note, the 5-year survival rate of patients treated with PD-1 alone could reach 35–40% (20–22).

Although the remarkable benefits of immunotherapy are evident, evidence from recent studies demonstrated that ICIs are associated with acquired or innate resistance (23). Immunotherapy focuses on harnessing the immune system to target and eradicate malignant cells. In melanoma, the level of infiltrating T cells correlate with immune response (24, 25). However, in most cases, the intro-tumoral immune response cannot be effectively activated due to the tumor microenvironment (TME) (26, 27). Notably, TME is an integral part of cancer forming an “ecosystem” to support tumor growth. It comprises numerous different cells and non-cellular factors at different stages of tumor development (28). TME is characterized by hypoxia and nutritional deficiency, i.e., conditions that limit the survival and function of effector T cells, but promote the formation of immunosuppressive cells, including myeloid-derived suppressor cell (MDSC), regulatory T cells (Tregs), and tumor-associated macrophages (29–31). Thus, understanding and targeting the TME is a promising approach for enhancing immunotherapy.

Herein, we performed a comprehensive analysis to explore immune infiltration in melanoma using four methods and constructed a prediction model. Consequently, we observed a higher infiltration and correlation of immune cells in metastatic tumors compared to primary tumors. Besides, patients with higher immune infiltration demonstrated a better survival. To uncover the underlying mechanisms of immune infiltration, the tumor was subdivided into hot and cold tumors then we calculated the DEGs between the two types of tumors. We found that TYRP1 was negatively correlated with CD8A expression while knockdown of TYRP1 promoted the expression of HLA-A, B, and C in tumor cells. Additionally, the prediction model performed efficiently in predicting the overall survival of patients with melanoma under immunotherapy.

## Methods and Materials

### Ethics Statement

Treatment naïve melanoma specimens were obtained after surgical treatment in the Second Affiliated Hospital of Zhengzhou University. All specimens were frozen in the biobank, and patients received conventional chemotherapy and were followed up every 6 months. All participants signed an informed consent form approved by the ethics committee of Second Affiliated Hospital of Zhengzhou University (Ethics number: 2020026).

### Cell Culture

Human melanoma cell line RPMI 1846 was purchased from the Chinese Academy of Sciences Cell Repertoire (Shanghai, China). Cells were cultured using a complete Roswell Park Memorial Institute (RPMI)-1640 medium containing 10% fetal bovine serum, 100 units/ml of penicillin, and 100 μg/ml of streptomycin (Thermo Fisher Scientific, USA) in a humidified incubator at 37°C with 5% CO_2_.

### Data Collection

The level 3 RNA-sequencing data and clinical information of skin cutaneous melanoma were downloaded from the online website UCSC Xena (https://xenabrowser.net/) (32), as in log2(x+1) transformed RSEM normalized count. Besides, the count data and survival information of metastatic urothelial cancercancer were downloaded from the platform supplied in the article (http://research-pub.gene.com/IMvigor210CoreBiologies/) (33). The RNA sequencing data and survival information of the melanoma were downloaded from the GEO database (https://www.ncbi.nlm.nih.gov/) with accession numbers: GSE78220, GSE91061 (9, 34).

### Immune Estimation

Further, ssGSEA (Single-Sample Gene Set Enrichment Analysis) was performed to derive the enrichment score of each immune-related term using an R package “GSVA” (35). Online web tool CIBERSORT (http://cibersort.stanford.edu/) algorithm was used to estimate the proportion of 22 immune cell types. Samples with CIBERSORT output *p*-value <0.05 were considered eligible for further analysis (36). The infiltrating level of CD4^+^ T cells, CD8^+^ T cells, B cells, macrophages, Dendritic Cells, and Neutrophils was downloaded from an online website, TIMER (https://cistrome.shinyapps.io/timer/) (37). The “MCP-Counter” package in R was used for analysis of microenvironment cell populations (MCPs) and quantification of immune cells (38). The immune score, stromal score, and tumor purity were calculated by R package “ESTIMATE.”

### Analysis of Differently Expressed Genes (DEGs)

Based on the immune score and immune cell infiltration, the samples were subdivided into two groups, i.e., hot and cold tumors. Subsequently, the differential expression analyses were conducted between the two groups using the R package “Limma,” with parameters of logFC >1.5 or <−0.5 and *p* value <0.05. Volcano diagram and heatmap were used to visualize the DEGs using R packages”ggplot2” and “pheatmap.”

### Enrichment Analysis and Protein-Protein Network Analysis

For the enrichment analysis, this study selected genes with p < 0.05 differently expressed in hot and cold tumors. R packages “clusterprofile” were used to analyze gene ontology (GO) terms and Kyoto Encyclopedia of Genes and Genomes (KEGG) enrichment analysis. P < 0.05 and q < 0.05 showed statistical significance. Genes with p < 0.05 and logFC > 2 and logFC < −0.5 were utilized to perform protein-protein interaction (PPI) network *via* online tool STRING (https://string-db.org/) with 0.9 confidence (39). For upregulated genes in PPI network, we used k means method to cluster the network. Nodes with less than two links were excluded for visualization.

### Consensus Clustering

The consensus clustering value method provides quantitative and visual stability evidence for estimating the number of unsupervised classes in a dataset (40). ConsensusClusterPlus implements the CC method in R extending it with new functionality and visualizations including item tracking, item-consensus, and cluster-consensus plots. Clustering was performed using the cluster Cons package with 100 iterations using a Manhattan distance metric then the most robust number of clusters was selected. The optimal number of clusters was established by the heat map and dela diagram.

### siRNA Transfections

The siRNA sequence was designed by BIODEV (http://biodev.cea.fr/DSIR/) and synthesized by Sangon Biotech (Shanghai, China). The sequence of si 1F: 5′-GGUCUUAACUACUAUGUUAUA-3′, R:5′-UAACAUAGUAGUUAAGACCAG-3′andsi2F:5′-GGUUCUGAUUAUUACGUUAAU-3′, R:5′-UAACGUAAUAAUCAGAACCUG-3′. Sangon Negative Control siRNA was used as control. The cells were seeded in an antibiotic-free complete medium at a density of 5 × 105 cells and cultured for 24 h. Transient transfection of cells with siRNA for 24 h was performed using Lipofectamine 2000 based on the manufacturer’s protocol. At 24 h after transfection, the medium was changed and the cells were allowed to recover.

### Quantitative Real-Time PCR

The fresh tumor specimens were obtained after the surgery then washed three times using PBS. Thereafter, tumor tissues were cut using scissors and added with Trizol (TaKaRa, Tokyo, Japan). To detect mRNA expression in tumor cell lines, tumor cells were transfected with siTYRP1 for 48 h then cells were obtained. The concentration and purity of total RNA were detected by NanoDrop 2000 (Thermo Fisher Scientific, MA, USA). Exactly 1 ug of total RNA was used to reverse into cDNA using ReverTra Ace qPCR RT Kit (TOYOBO, OSAKA, Japan). The primers used were designed and purchased from Sangon Biotech (Shanghai, China) (**Supplementary Table 1**). Glyceraldehyde-3-phosphate dehydrogenase (GAPDH) was used as control.

### Correlation Analysis

The spearman correlation of immune cells was performed by R packages,”ggcor.” The spearman correlation of CD8A expression and TYRP1 expression in the TCGA database and clinical samples was performed by GraphPadPrism (version 7.00).

### Construction of Prediction Model

The RNA-sequencing data with survival information of melanoma was randomly divided into training and testing cohort by R package “caret.” Genes differently expressed in hot and cold tumors were used to perform univariate survival analysis, and genes with p < 0.05 were selected. Then, R packages”glmnet” was used to perform LASSO analysis with maix = 20,000. To optimize the model, this work used a step-wise proportional hazards model. The survival analysis was analyzed by R package “survival,” while AUC was analyzed by R package” survivalROC.” To validate the model, clinical samples with survival information were obtained and the expression of genes in the model was calculated by RT-PCR. The RNA-sequencing data and survival information of patients under immunotherapy treatment were obtained from the GEO database or supplied in the article. The count and FPKM data were transferred into TPM and made a log2(x+1) normalization.

### Statistical Analysis

All analyses were performed using R version 3.6.1. WilcoxTest was used to compare the infiltration of immune cells in primary and metastatic melanoma, as well as in hot and cold tumors, whereas ANOVA was used to compare immune score, stromal score, and tumor purity among the four clusters. For the survival analysis, the p-value was calculated with a log-rank test. In all analyses, P < 0.05 was considered statistically significant.

## Results

### Infiltration Patterns Between Primary and Metastatic Melanoma

**Figure 1:**
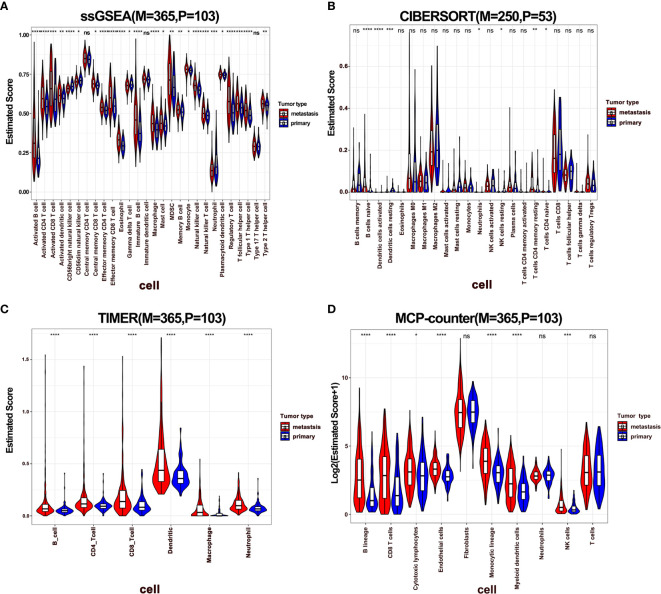
The difference of immune infiltration between primary and metastatic tumors. **(A–D)** Violin plot showed the score of immune cells estimated by ssGSEA, CIBERSORT, TIMER, and MCP-Counter. P represents primary, M represents metastatic.

To dissect the difference of the immune infiltration between primary and metastatic melanoma, the proportion of immune cells was calculated using four different methods, including ssGSEA, CIBERSORT, TIMER, and MCP-Counter. The four methods exhibited various algorithms focusing on different sets of immune cells. ssGSEA, TIMER, and MCP-Counter demonstrated consistent results, while CIBERSORT results differed from others (**Figures 1A–D**). The proportion of the most of immune-related cells in metastatic melanoma was higher than in primary melanoma, specifically B cells, CD4^+^ T cells, CD8^+^ T cells, and DCs. These findings indicate a stronger immune response in metastatic melanoma compared to primary melanoma. Also, a few immune immunosuppressive cells were enriched in metastatic tumors, including myeloid-derived suppressive cells (MDSCs), regulatory T cells (Tregs). Moreover, neutrophils and CD56 bright natural killer (NK) cells were enriched in primary melanoma, indicating the significance of innate immunity in primary tumors. Additionally, a higher infiltration of mast cells was noted in metastatic tumors (**Figures 1A, B**). Notably, mast cells exhibit an important role in connecting innate and adaptive immunity, but also with pro-tumor function in TME (41). Nevertheless, its role in metastatic melanoma remains unclear hence warrants further investigation.

### Correlation of Immune Cells in Primary and Metastatic Melanoma

**Figure 2:**
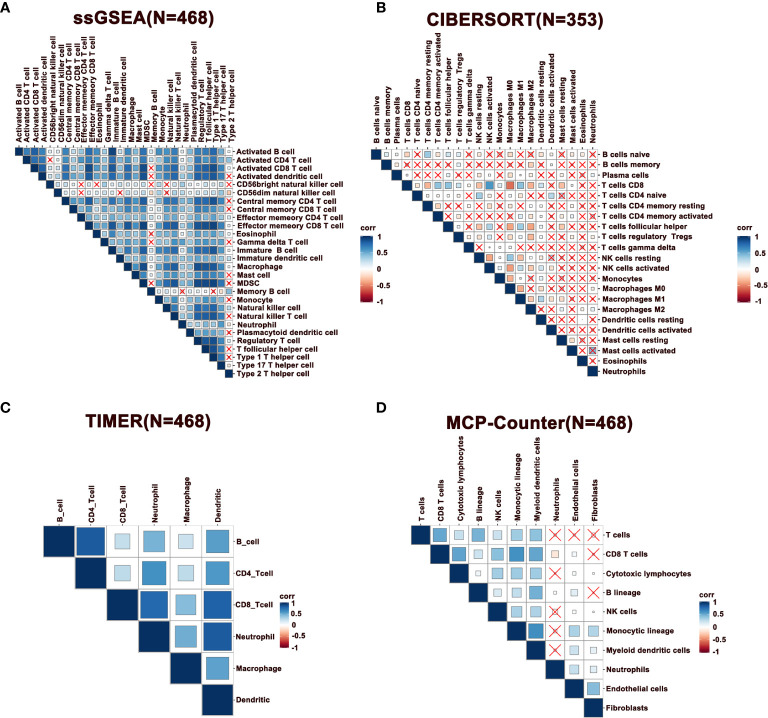
Correlation of immune cells in melanoma. **(A–D)** Spearman correlation analysis of immune cells estimated by ssGSEA, CIBERSORT, TIMER, and MCP-Counter. N represents number of patients.

Notably, immune infiltration requires synergic activity of multiple cells in tumor tissues. To this end, we performed a correlation analysis of immune cells in melanoma, where the results of ssGSEA, TIMER, and MCP-Counter revealed that immune cells exhibited a relatively strong correlation (**Figures 2A, C, D**). However, this phenomenon was not observed with the immune cells estimated by the CIBERSORT method (**Figure 2B**). Specifically, CD8^+^ T cells were positively correlated with follicular helper T cells (Tfhs), activated NK, and DCs, indicating a cooperation across these cells in immune response. Further, it was observed that activated CD8^+^ and CD4^+^ T cells were positively correlated with macrophages M1, while negatively associated with M0 and M2 macrophages, demonstrating that M1 exhibits the function of antigen presentation (**Figure 2B**). Besides, neutrophils were negatively correlated with T cells. Furthermore, a correlation analysis was performed in metastatic melanoma and primary melanoma, respectively. Consequently, a higher correlation of immune cells was discovered in metastatic tumors compared to primary tumors (**Supplementary Figures 1A–D**).

### Immune Subtyping of Melanoma

**Figure 3:**
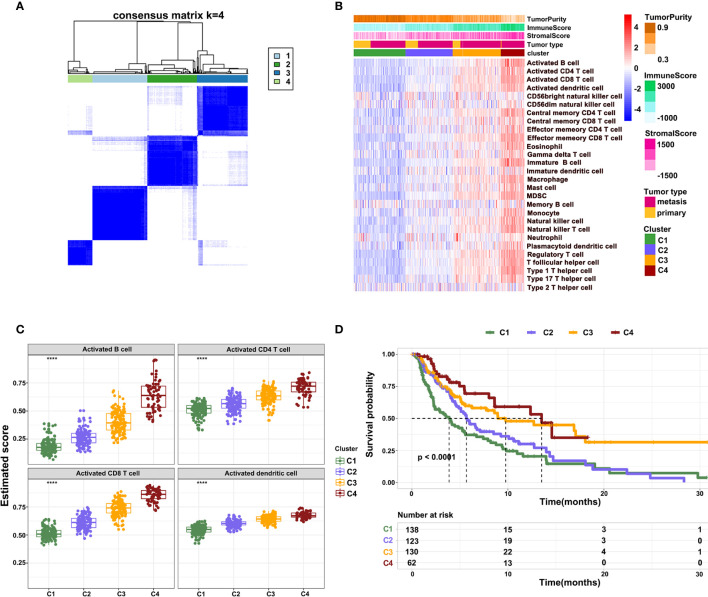
Different patterns of immune cell infiltration in normal and tumor tissue. **(A)** Heatmap showing the consensus clustering of immune cells. **(B)** Heatmap showing the distribution of 28 immune cells across four clusters. **(C)** The infiltration of activated B cells, CD4^+^ T cells, CD8^+^ T cells, and DCs across four clusters. **(D)** Kaplan-Meier survival curve showing overall survival of patients across four clusters.

To further characterize the immune infiltration in melanoma, we performed a consensus clustering analysis of immune cells calculated by ssGSEA. The heatmap revealed that the melanoma could be divided into four clusters (**Figure 3A**). From clusters 1 to 4, there was a gradual increase of immune infiltration in tumor tissue. Cluster1 lacked infiltration of immune cells, clusters 2 and 3 had modest infiltration of immune cells, while cluster 4 showed a phenotype of abundant immune infiltration. This was also reflected by the immune and stromal scores across four clusters (**Figure 3B**). Consistently, activated B cells, CD4^+^ T cells, CD8^+^ T cells, and DCs had the highest score in cluster 4 (**Figure 3C**). Notably, a few innate immune cells, including NK cells and neutrophils, showed no changes in four clusters. On other hand, clusters 3 and 4 had high infiltration of Tregs, MDSCs, and immature DCs, which inhibited immune response in tumors. These findings potentially suggest that immune activation is also accompanied with immune suppression mediated by the TME. Survival analysis revealed that clusters 3 and 4 had better survival relative to clusters 1 and 2 (**Figure 3D**). These outcomes indicate that the degree of immune infiltration positively correlates with patient survival.

### The Difference of Immune-Related Genes in Four Clusters of Melanoma

**Figure 4:**
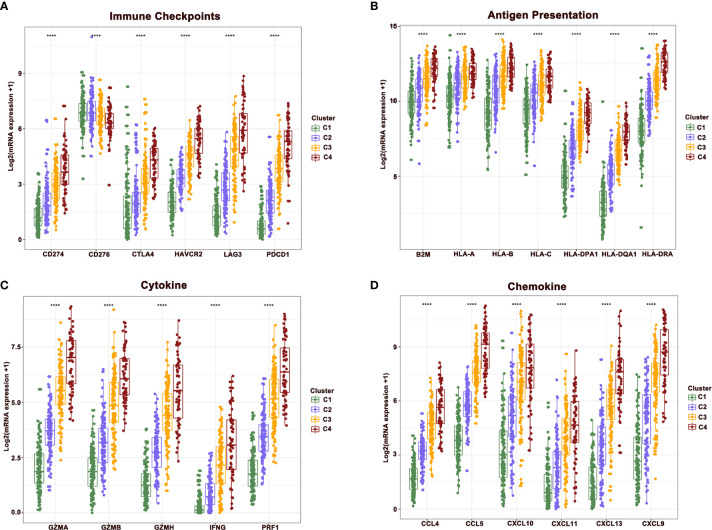
The difference of central immune molecules in melanoma. **(A–D)** The expression of immune checkpoint, antigen presentation, cytokine and chemokine-related genes across four clusters.

To further reveal the mechanisms of cellular immunity among the four clusters, the expression of immune checkpoints, antigen presentation, cytokines, and chemokine-related genes were analyzed in four clusters. Results revealed that these molecules showed a higher expression in clusters 4 and 3 relative to clusters 1 and 2. Nevertheless, the expression of CD276 gradually showed the opposite trend, being highly expressed in clusters 1 and 2, while downregulated in clusters 3 and 4. The mutually exclusive expression pattern of CD276 and other immune checkpoints might provide potential treatment options for patients who do not respond to anti-PD-1, PD-L1, and CTLA-4 treatment (**Figures 4A–D**).

### Alterations of Signaling in Hot and Cold Tumor

**Figure 5:**
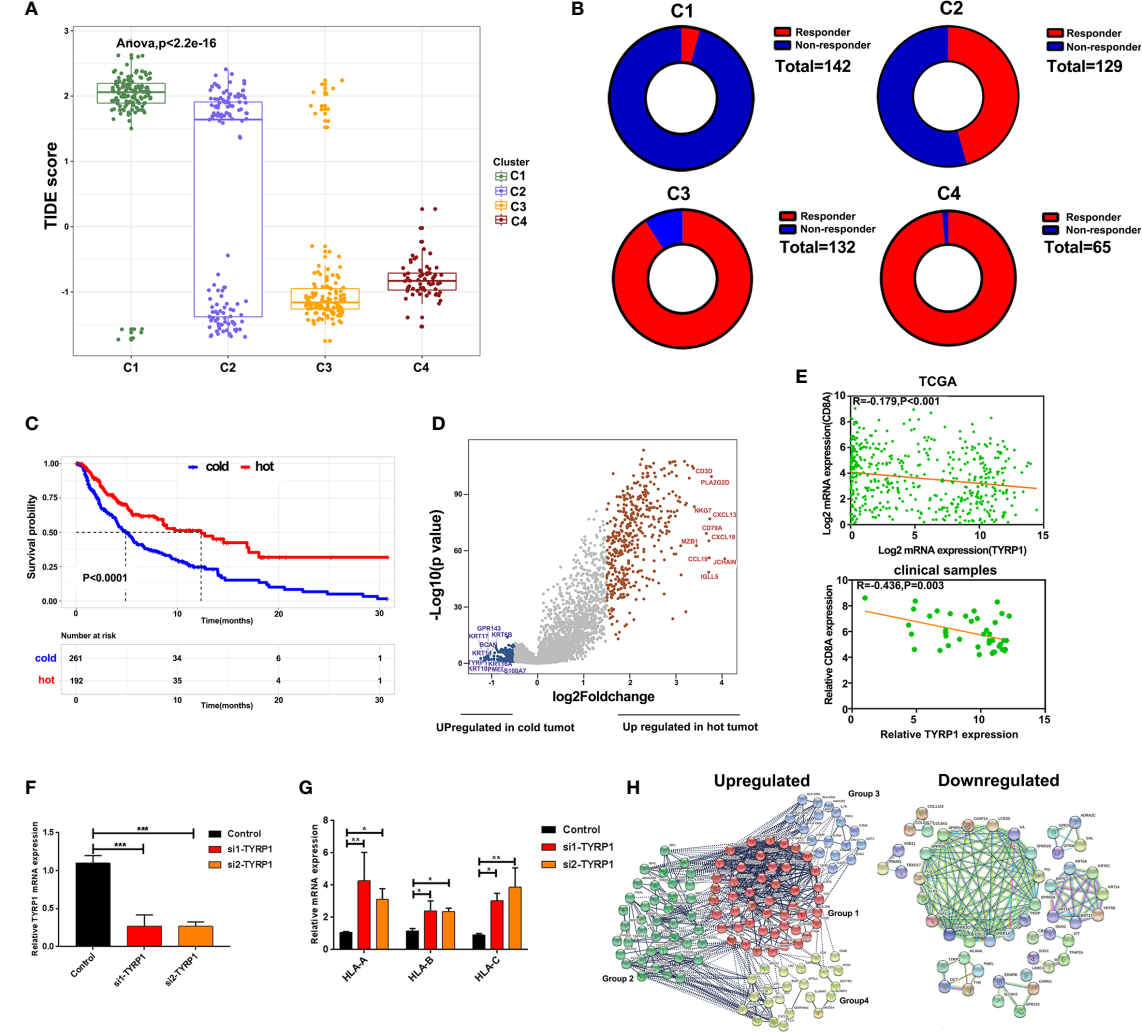
Alterations of signaling in hot and cold tumor. **(A)** Boxplot showed the TIDE score of four clusters. **(B)** Ring plot showed the response rate of immunotherapy across four clusters. **(C)** Kaplan-Meier survival curve showed the survival of patients in hot and cold tumor. **(D)** Volcano showing DEGs between hot and cold tumor. **(E)** Correlation analysis of TYRP1 in TCGA (upper) and clinical samples (lower). **(F)** RT-PCR analyzed the efficiency of knockdown FABP6. **(G)** RT-PCR analyzed the expression of HLA-A, B, and C transfected with si-TYRP1. **(H)** The PPI network of DEGs in hot tumor and cold tumor.

To predict the response of four clusters to immunotherapy, an online website Tumor Immune Dysfunction and Exclusion(TIDE) was used to calculate the tumor immune dysfunction and exclusion score. As a consequence, clusters 4 and 3 had significantly lower scores compared to clusters 1 and 2 (**Figure 5A**). In line with these findings, clusters 4 and 3 had a higher rate of responders to immunotherapy estimated by TIDE (**Figure 5B**). To further explore the mechanisms of immune infiltration, melanoma was subdivided into two subtypes, i.e., hot and cold tumor, with hot tumor comprising clusters 3 and 4, while cold tumor comprising clusters 1 and 2. Survival analysis revealed that hot tumor was expected to exhibit a better survival (**Figure 5C**). Subsequently, the difference between the two types of tumors was analyzed at the transcription level. Hot and cold tumors demonstrated distinct transcription patterns as illustrated by the volcano map and heatmap (**Figures 5D** and **Supplementary Figure 2**). The top 10 DEGs between hot and cold tumors were marked in the volcano map. Several genes related to immune activation enriched in hot tumors have been reported, including CD3D, PLA2G2D, NKG7, CXCL13, CD79A, and CXCL9. Additionally, tyrosinase-related protein1 (TYRP1) was upregulated in cold tumors and negatively correlated with CD8A expression (**Figure 5E**). *In vitro* experiments revealed that knockdown TYRP1 promoted the expression of HLA-A, B, and C. These findings indicate that inhibition of TYRP1 potentially promote the antigen presentation of MHC class I in tumor cells (**Figures 5F, G**). To investigate the interactions of DEGs, a PPI network of DEGs was performed. As a consequence, the PPI network in hot tumors formed four groups. Group 1 comprised genes in HLA families and B cell lineage, such as FCER1G, FCGR3A, CD79, CD19, which represent antigen presentation. Group 2 were genes implicated in T cell stimulation and recruitment, etc. Group 3 included cytokine and exhausted-related genes, such as GZMB, PRF1, HAVCR2, etc. Group 4 also contained several T cells-related chemokines and activated genes, including CCL4, CXCL9, SLAMF1, etc. The PPI network in cold tumor comprises a cluster of small proline-rich proteins (SPRR) family, while its role in melanoma is unclear (**Figures 5H**).

### GO and KEGG Pathway Enrichment Analysis of DEGs

**Figure 6:**
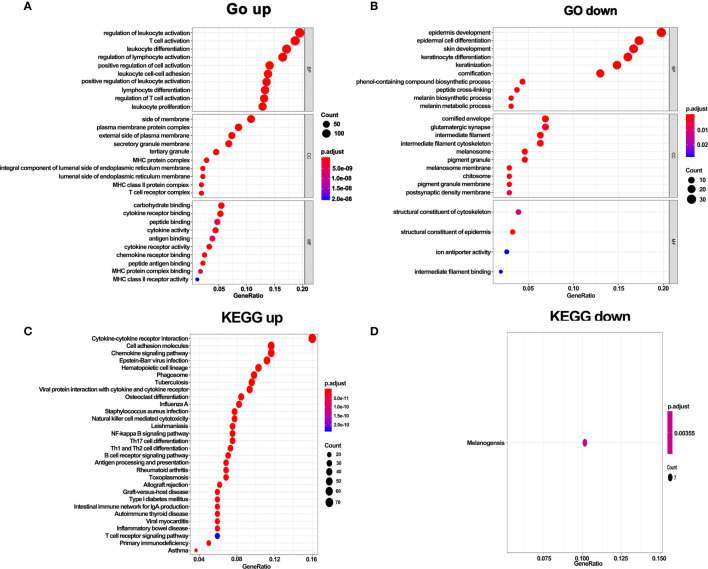
GO terms and KEGG enrichment analysis of DEGs. **(A)** GO enrichment analysis of upregulate DEGs. **(B)** GO enrichment analysis of downregulate DEGs. **(C)** KEGG pathway enrichment analysis of upregulate DEGs. **(D)** KEGG pathway enrichment analysis of downregulate DEGs.

To further explore the function of DEGs, GO and KEGG enrichment analyses were performed. In line with the above results, GO analysis revealed that DEGs in hot tumors were primarily enriched in the regulation of leukocyte activation and leukocyte cell-cell adhesion. On the other hand, DEGs in cold tumors were significantly enriched in epidermis development, epidermal cell keratinocyte differentiation, structural constituent of cytoskeleton, structural constituent of epidermis, and ion antiporter activity. These outcomes suggest that the immunity was comprehensively activated in hot tumors, particularly leukocyte-mediated immune responses. In contrast, it was inclined to form a tough structure, including cytoskeleton and cornified envelope in cold tumor, which might prevent the infiltration of immune cells (**Figures 6A, B**). Moreover, the results of KEGG pathways enrichment analysis confirmed that activated immune response was observed in hot tumors (**Figure 6C**). Notably, the KEGG pathway in cold tumor was melanogenesis (**Figure 6D**).

### Construction and Validation of Prediction Model for Overall Survival

**Figure 7:**
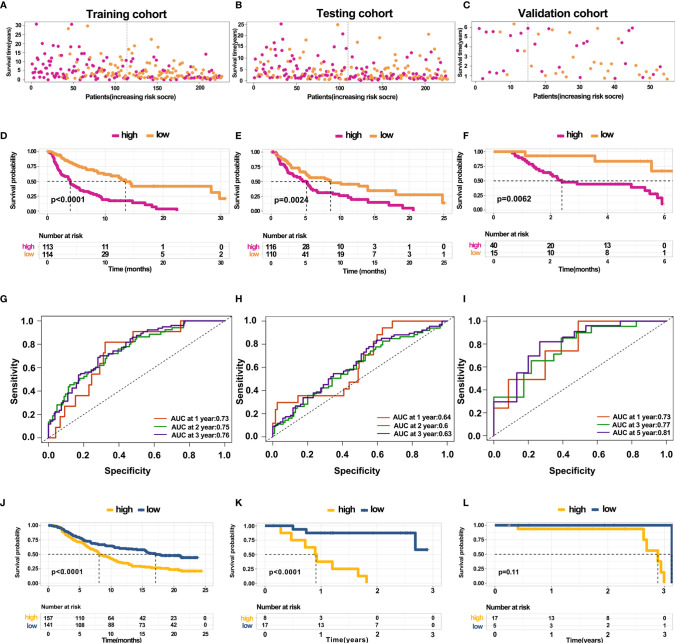
Construction and validation of predicting model for overall survival **(A–C)** Distribution of survival time in the training cohort, testing cohort, and external validation cohort. **(D–F)** Kaplan-Meier survival curve showed the survival of patients with high and low risk in the training cohort, testing cohort, and external validation cohort. **(G–I)** AUC curve of 1, 3, and 5 years for training cohort, testing cohort, and external validation cohort. **(J–L)** Kaplan-Meier survival curve showed the survival of patients under immunotherapy treatment with low and high risk in bladder cancer and melanoma.

The above results revealed that immune infiltration was correlated with the survival of patients. Therefore, the value of DEGs in predicting the overall survival of melanoma patients in TCGA and our external validation cohort should be explored. The detailed information of patients in TCGA and our clinical samples is shown in **Table 1**. Based on equal mortality rates, patients from the TCGA dataset were randomly divided into training and testing cohorts. Then, a LASSO regression model was used to identify the best gene sets for predicting OS in the training cohort (**Supplementary Figures 3A, B**). To optimize the model, a stepwise multi-Cox regression model was performed to select the most predictive gene sets (**Supplementary Figure 3C**). Eventually, a gene set containing seven genes was identified; where six of seven (CALHM1, OCSTAMP, HRASLS2, CEBPB, ICAM1, IFITM1) genes were upregulated in the low-risk group, one of seven (TTYH3) was upregulated in the high-risk group (**Supplementary Figures 4A–C**). Then, a risk value was calculated based on the expression levels of selected genes and the corresponding regression coefficients: Risk score = 0.2577 × TTYH3 expression −3.3455 × CALHM1 expression − 1.3156 × OCSTAMP expression − 0.7372 × HRASLS2 expression − 0.2322 × CEBPB expression − 0.1963 × ICAM1 expression − 0.1219 × IFITM1 expression in three cohorts (**Supplementary Figures 4D–F**). Results revealed that the risk score effectively distinguished the survival time of patients in training, testing, and validating cohorts (**Figures 7A–C**). Consistent with these findings, patients with high risk predicted a poor survival (**Figures 7D–F**). The AUC of the predicting model for the training dataset at 1^st^ year, 2^nd^ year, and 3^rd^ year was 0.73, 0.76, 0.75, while 0.64, 0.6, and 0.63 for the testing cohort (**Figures 7G, H**). The AUC in the validating cohort were 0.73, 0.77, and 0.81 at 1^st^, 2^nd^, and 3^rd^ year, respectively, indicating a satisfactory value for predicting the overall survival of patients (**Figure 7I**). For further analysis, the seven gene signatures were subsequently evaluated in predicting survival of patients receiving immunotherapy in three independent cohorts, where one was metastatic urothelial cancer cancer and the other two were melanoma. Results revealed that patients with high risk had an unfavorable overall survival in two cohorts, showing no significant difference in a melanoma cohort (**Figures 7J–L**). Generally, these findings suggest that the prediction model performed efficiently in predicting overall survival and can guide the clinical treatment of patients with melanoma.

**Table 1:** Clinical and pathologic characteristics of the patients in TCGA and external validation cohort analyzed in this study.

Characteristics	TCGA	Validation cohort
Number of samples	453	60
Age median (range)	58 (15–90)	53 (29–73)
Gender		
Male	282 (62%)	36 (60%)
Female	171 (38%)	24 (40%)
Additional_pharmaceutical_therapy		
Yes	32 (7%)	39 (65%)
NO	32 (7%)	21 (35%)
NA	389 (86%)	
Additional_radiation_therapy		
Yes	46 (10%)	8 (13%)
NO	19 (4%)	52 (87%)
NA	388 (86%)	
Pathologic_M		
M0	403 (89%)	50 (83%)
M1	24 (5%)	10 (17%)
NA	26 (6%)	
Pathologic_N		
N0	221 (49%)	32 (53%)
N1	73 (16%)	18 (30%)
N2	49 (11%)	6 (10%)
N3	56 (12%)	4 (7%)
NX	35 (8%)	
NA	19 (4%)	
Pathologic_T		
T0	23 (5%)	
T1	41 (9%)	14 (23%)
T2	76 (17%)	28 (47%)
T3	89 (20%)	13 (22%)
T4	152 (34%)	5 (8%)
TX	45 (10%)	
NA	27 (6%)	
Pathologic_Stage		
Stage 1	86 (19%)	19 (32%)
Stage 2	138 (30%)	21 (35%)
Stage 3	170 (38%)	14 (23%)
Stage 4	23 (5%)	6 (10%)
NA	36 (8%)	

## Discussion

Melanoma is evolving as the most threatening form of skin tumor with its global incidence rapidly increasing. In the early stages, surgery is the effective treatment option for melanoma where the survival rates are significantly high, however, they significantly drop after metastasis. Reports indicate that the median overall survival of metastatic melanoma was less than 1 year (42). Early misdiagnosis of melanoma minimizes the survival of melanoma patients, causing metastasis which accounts for the majority of mortalities (43). Considering tumor recurrence and resistance manifesting within a relatively short time for most patients (44, 45) and elevated mutation load of melanoma (46), the treatment with new drug combinations has become a vital strategy in achieving a sustainable effect. Data from clinical trials revealed that advanced melanoma patients treated with ICIs alone or in combination prolonged the survival and demonstrated a higher objective response rate (20). Nonetheless, accumulated data reveals several patients with zero response to treatment, and a few patients initially responding to treatment but eventually develop resistance due to the complex TME (47). As such, an in-depth understanding of immune status in melanoma is essential to guide its clinical treatment.

This study used four methods to investigate the immune infiltration in melanoma. Specifically, we compared the immune infiltration in primary and metastatic melanoma, respectively, as a result, metastatic melanoma showed a higher immune infiltration. Also, previous studies demonstrated that metastatic melanoma is considered a perfect example of an immunogenic tumor since it is characterized by the consistent presence of lymphoid infiltrate (48). While, most of cells estimated by these four methods are different, although same cells are belong to the same cell linage, such as T cell lineage (CD4 and CD8^+^ T cells), B cell lineage, and macrophage lineage. For a single cell type, it may be difficult to draw a consistent conclusion from these four methods. But different cell types also provide more information for exploring the heterogeneity of the tumor microenvironment. Meanwhile, a strong correlation of immune cells was noted in metastatic melanoma. Besides, mast cells were enriched in metastatic tumors as showed by ssGSEA. Notably, mast cells play critical roles in both innate and adaptive immunity producing large subsets of mediators and reshaping the tumor microenvironment of melanoma. Several case reports and studies have confirmed an enhanced incidence of melanoma among patients with mastocytosis (49–51). Mast cells act as pro-tumor in the TME (52–54). Targeting mast cells infiltrated in TME combined with immune checkpoint inhibitors or turning tumor-promoting mast cells into tumor-inhibiting mast cells might be an effective approach for the treatment of melanoma (55). Here, we calculated the relative expression level of immune cells in each sample using CIBERSORT. Notably, the immune levels estimated by CIBERSORT were not similar to other methods. This method effectively reflects the ratios of cells in each sample but it cannot represent the absolute number of cells. The CIBERSORT algorithm considers a signature matrix built from microarray data, comprising 22 immune cell types, and estimates the cell fractions using nu support vector regression (36). Unlike CIBERSORT, TIMER estimates six immune cell types based on immune-specific markers. However, TIMER cannot used to compare across different cell types (56), and both of the two methods use deconvolution. ssGSEA and MCP-counter calculate score based on marker genes and ranks the genes *via* their absolute expression in a sample and computes enrichment score for each cell type. This method has been widely used since individual gene sets can be defined. MCP-counter is computed as the geometric mean of the expression values of cell-type-specific genes. Different methods have their advantages and disadvantages. CIBERSORT focuses on the ratios of each cell in each sample, thus, cell comparisons between different samples may bring a relatively large deviation. The data analyzed by CIBERSORT can be microarray and RNA-seq, but for RNAseq, the data in Transcripts Per Kilobase of exonmodel per Million mapped reads (TPM) format is more accurate. Although the online webtool TIMER only score six cell types, the markers used in this method contain specific genes in each tumor. Therefore, it may be more accurate to assess the degree of cell infiltration. ssGSEA has fewer restrictions on the format of the data, and the calculated cell score can be used for comparison of different samples. At the same time, it can be more flexible to calculate the cell composition in the microenvironment based on the accurate marker gene (35, 38). Therefore ssGSEA may be a better choice for most sequencing data. Nevertheless, among the above, an effective method remains controversial.

Several distinguishable subtypes of melanoma from clinical and pathology have been reported. Also, studies have reported that these subtypes usually exhibit distinct genetic characteristics in molecular biology (5). Recent empirical studies and reviews have revealed that the efficacy of immune checkpoint inhibitors differ depending on the subtype of melanoma. Moreover, studies have found that tumors with a higher mutation burden usually obtain more benefit from immune checkpoint inhibitors.

This paper clustered melanoma samples based on immune infiltration, and consequently, patients with higher immune response demonstrated better survival, affirming our definition of clusters. Additionally, immune-related genes were upregulated in clusters 4 and 3 relative to clusters 1 and 2, however, this did not include CD276. CD276, known as B7-H3, belongs to the B7 family of immunoregulatory proteins and has been implicated in cancer progression and metastasis. Research has confirmed the expression of CD276 in primary and metastatic melanoma as well as its significant role in the progression of melanoma and events of metastasis (57, 58). Additionally, our findings suggest that CD276 might serve as a potential target for patients with zero response to immunotherapy.

TIDE is a computational method that predicts the outcome of tumor patients treated with anti-PD1 or anti-CTLA4 and calculates the score by evaluating the degree of T cell dysfunction (59). The patients with a high score of TIDE imply a high possibility of tumor immune evasion and lower benefits from immunotherapy. In line with immune states across all the four clusters, clusters 4 and 3 had lower scores compared to clusters 1 and 2. Notably, the TIDE score of cluster 1, cluster 2, and cluster 3 had two clear subsets, particularly in cluster 2, indicating that these clusters still exits distinct subtypes and need further exploration. To explore the mechanisms of immune infiltration, the four clusters were divided into two major types, i.e., cold and hot tumors. We found that TYRP1 negatively correlated with CD8A expression. *In vitro* experiments revealed that the knockdown of TYRP1 promoted the expression of MHC class I. TYRP1 correlated with the formation of melanogenesis and was a cancer antigen of melanoma (60). Previous studies demonstrated that TYRP1 is linked to a poor clinical outcome for patients diagnosed with metastatic melanoma (61). Besides, it acts as a biomarker candidate for response and survival to checkpoint inhibitors in melanoma patients (62). Nonetheless, the mechanism of TYRP1 in regulating the immune response warrants further investigation.

Eventually, a prediction model based on the DEGs between hot and cold tumors was constructed, and this model could predict the overall survival of patients with treatment naïve melanoma. The model comprises seven genes which can be easily detected and guide the clinical treatment of melanoma. Notably, we found that this model performed efficiently in predicting the survival of patients under immunotherapy. However, the model has a few limitations; first, we only used one data set as an external validation cohort with a limited number of patients. Secondly, an optimal cut-off of risk score was used to divide the patients into high and low-risk scores, potentially reducing the predictive performance of the model. Thirdly, this model did not apply to melanoma patients under all types of therapies.

## Conclusion

In this conclusion, we performed a detailed analysis of immune infiltration in melanoma. Our findings revealed that patients with high immune infiltration had a better survival and immune response. Moreover, the knockdown of TYRP1 promoted the expression of MHC class I and serving as a potential target. We constructed a prediction model with seven genes, which performed effectively in predicting survival of treatment naïve melanoma patients or untreated with anti-PD1. This work provides an in-depth insight into immune infiltration and the prediction model can used to guide the treatment of patients with melanoma.

## Data Availability Statement

Publicly available datasets were analyzed in this study. This data can be found here: (https://xenabrowser.net/); GSE78220, GSE91061.

## Author Contributions

GL designed the experiments. GL and XZ performed the experiments and analyzed the data. XZ and CL conceived the work, analyzed the data, and prepared the manuscript. All authors critically revised the manuscript and agreed to be accountable for all aspects of the manuscript. All authors contributed to the article and approved the submitted version.

## Conflict of Interest

The authors declare that the research was conducted in the absence of any commercial or financial relationships that could be construed as a potential conflict of interest.

## Supplementary Material

Supplementary Figure 1 Correlation of immune cells in primary and metastatic melanoma respectively. **(A–D)** Correlation analysis of immune cells in primary and metastatic tumor estimated by ssGSEA, CIBERSORT, TIMER, and MCP-Counter.

Supplementary Figure 2 Heatmap showing the DEGs between hot and cold tumor.

Supplementary Figure 3 Identification of prognostic related genes. **(A, B)** LASSO and partial likelihood deviance coefficient profiles of survival-related genes analyzed by univariate survival analysis. **(C)** Frost plots showed the HRs of selected genes by multivariate Cox analysis.

Supplementary Figure 4 Construction and validation of predicting model. The expression of selected genes in high and low risk group in training cohort, testing cohort, and external validation cohort.
