# Supplementary figures and images for: Characterization of Immune Infiltration and Construction of a Prediction Model for Overall Survival in Melanoma Patients
Source: Front Oncol. 2021 Apr 2;11:639059. doi: 10.3389/fonc.2021.639059 (PMC8051586; doi:10.3389/fonc.2021.639059)

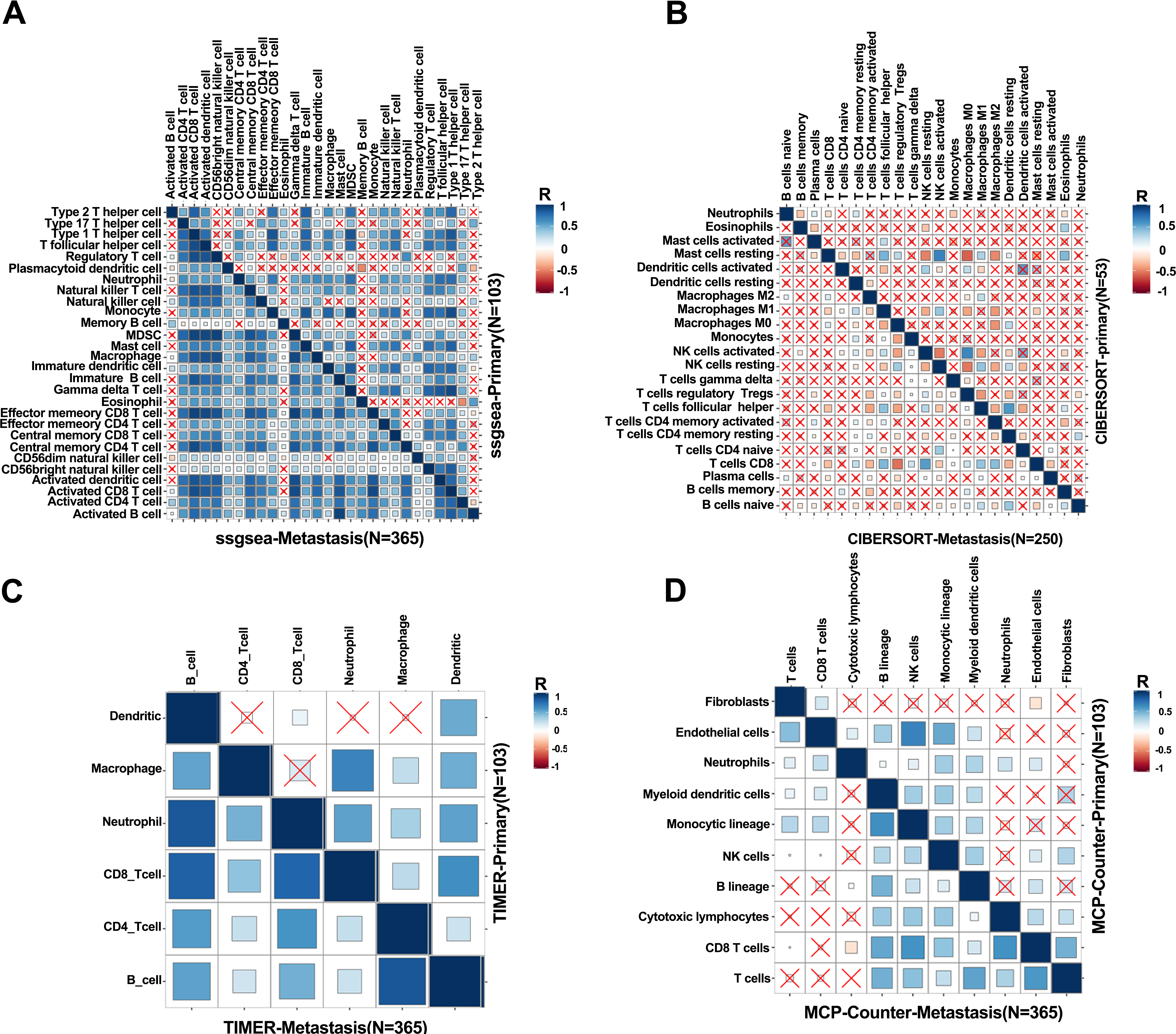

Supplement: Supplementary Figure 1 — Correlation of immune cells in primary and metastatic melanoma respectively. (A–D) Correlation analysis of immune cells in primary and metastatic tumor estimated by ssGSEA, CIBERSORT, TIMER, and MCP-Counter. [file Image_1.jpeg]

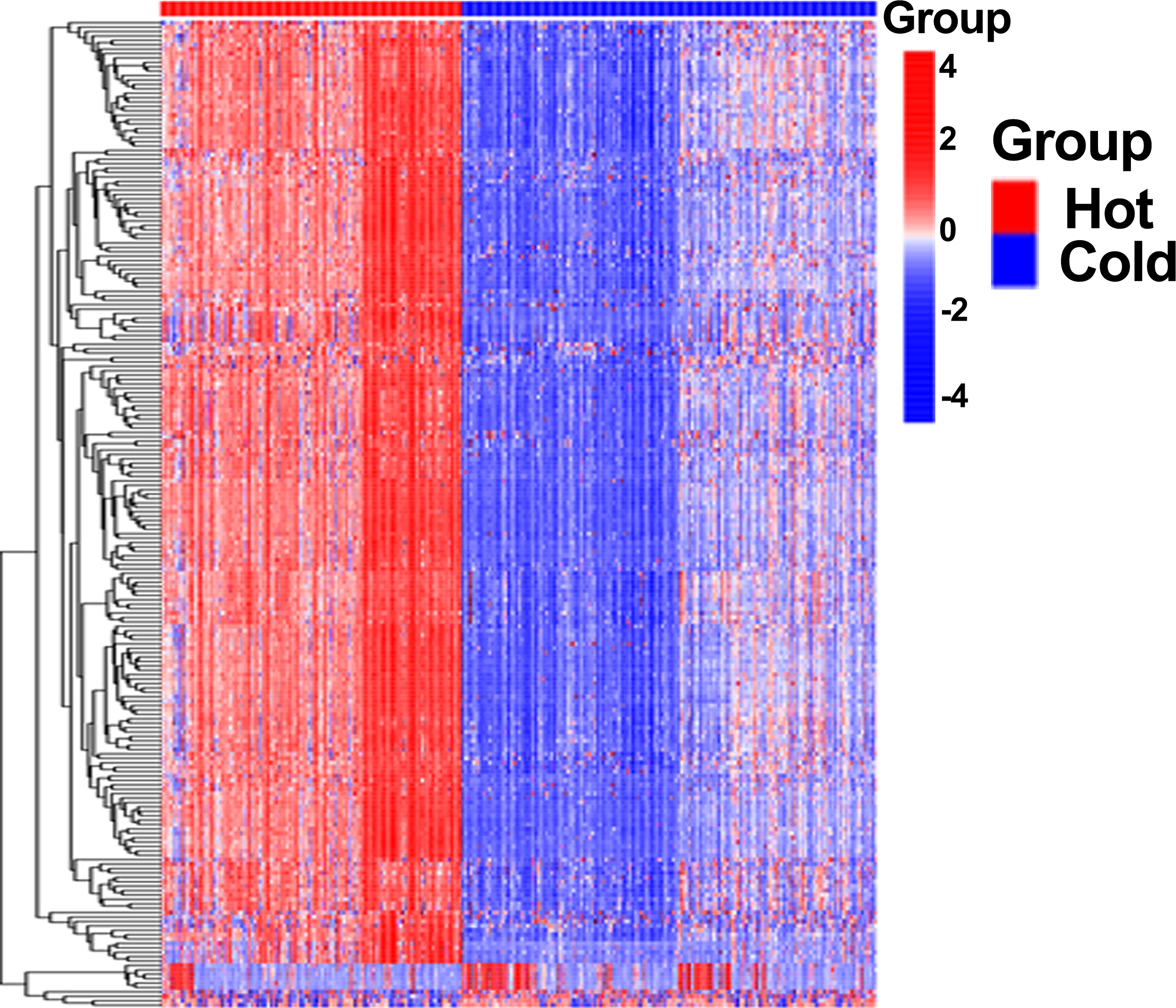

Supplement: Supplementary Figure 2 — Heatmap showing the DEGs between hot and cold tumor. [file Image_2.jpeg]

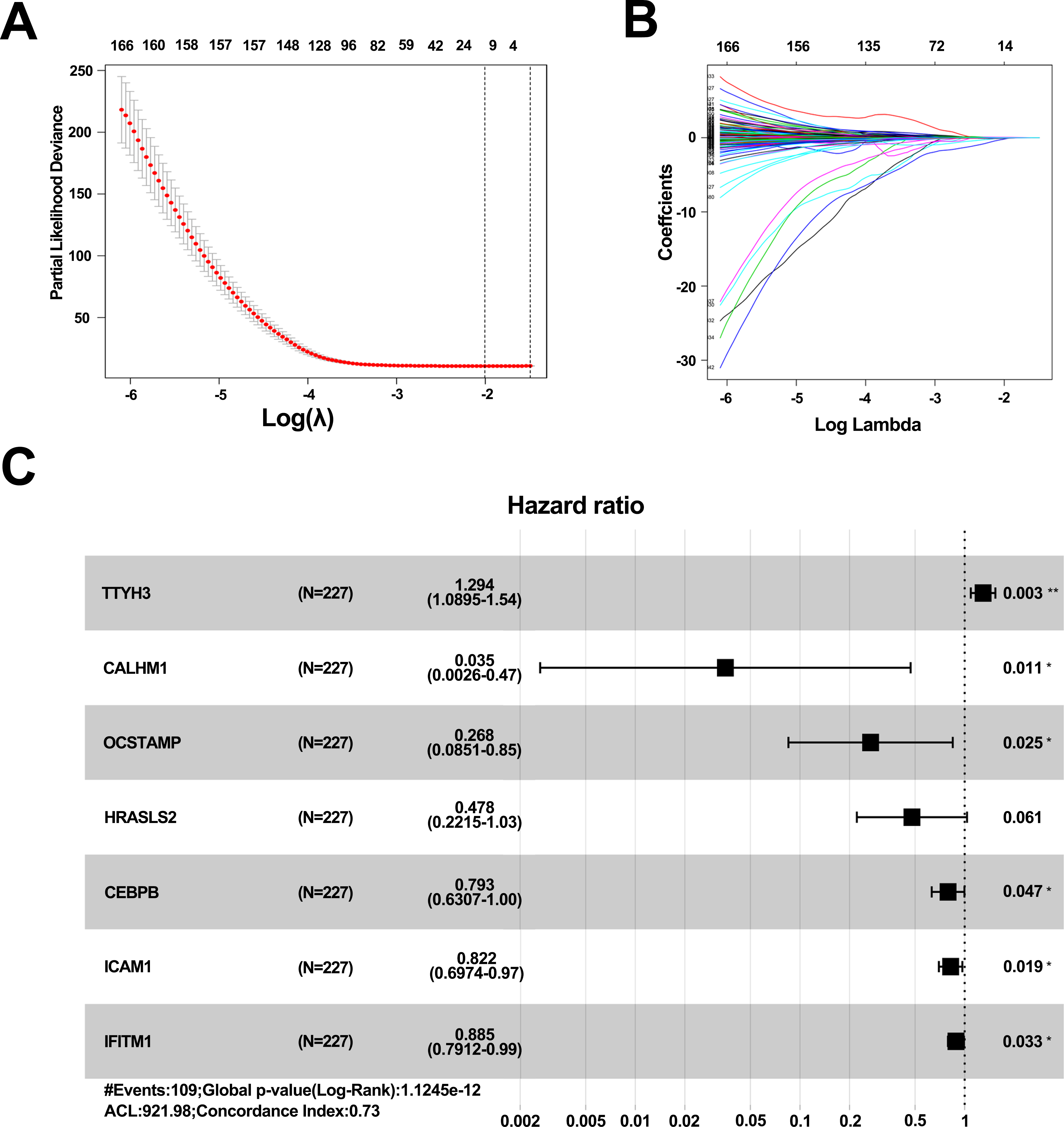

Supplement: Supplementary Figure 3 — Identification of prognostic related genes. (A, B) LASSO and partial likelihood deviance coefficient profiles of survival-related genes analyzed by univariate survival analysis. (C) Frost plots showed the HRs of selected genes by multivariate Cox analysis. [file Image_3.jpeg]

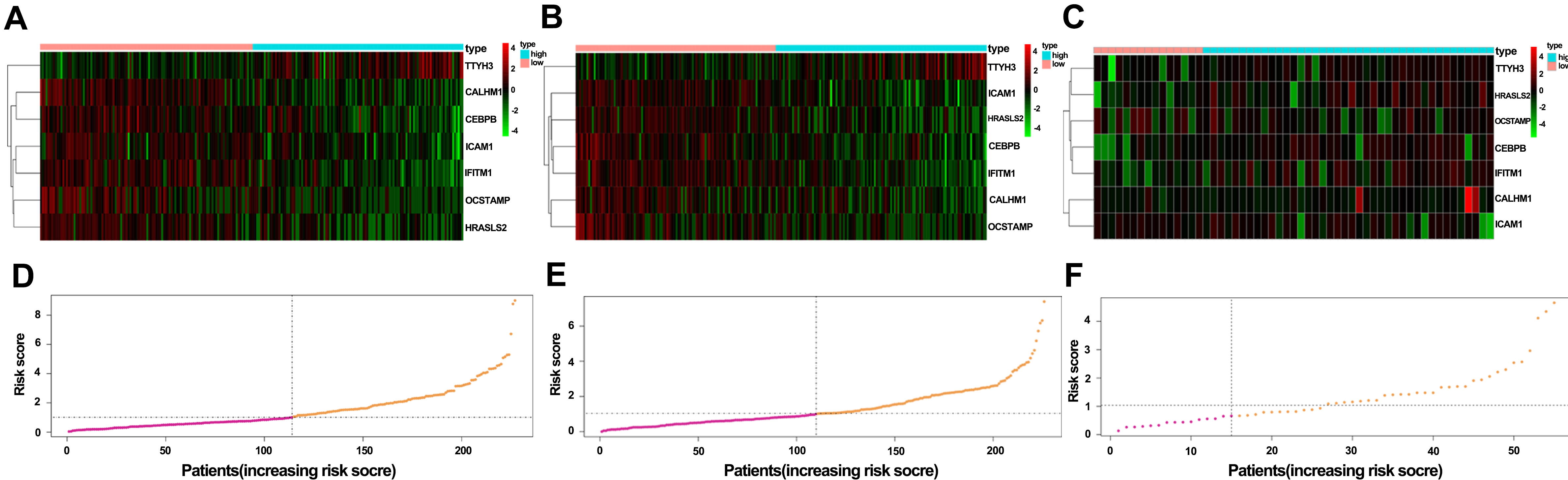

Supplement: Supplementary Figure 4 — Construction and validation of predicting model. The expression of selected genes in high and low risk group in training cohort, testing cohort, and external validation cohort. [file Image_4.jpeg]
