# Supplementary material for: Characterization of Immune Infiltration and Construction of a Prediction Model for Overall Survival in Melanoma Patients
Source: Front Oncol. 2021 Apr 2;11:639059. doi: 10.3389/fonc.2021.639059 (PMC8051586; doi:10.3389/fonc.2021.639059)
Supplement: Supplementary file 5 [file Table_1.docx]

| **Gene** | **Forward Primer** | **Reverse Primer** |
| --- | --- | --- |
| CD8A | ATGGCCTTACCAGTGACCG | AGGTTCCAGGTCCGATCCAG |
| TYRP1 | TCTCTGGGCTGTATCTTCTTCC | GTCTGGGCAACACATACCACT |
| TTYH3 | CGAGGACACCGACTACCAG | GTGGCGATGATGACACACCA |
| CALHM1 | CCAGTGCCCAGATGTACTCG | GCATGGACACGTTGTTGTTCA |
| OCSTAMP | CACCCTGGGTATGGAGCAG | CTGGTGAGTGGTATTGAGGAGA |
| HRASLS2 | GATTGAGATTTCTCGCTTTGGCT | CGTGCTTGTTATTGACCCTGTA |
| CEBPB | CTTCAGCCCGTACCTGGAG | GGAGAGGAAGTCGTGGTGC |
| ICAM1 | ATGCCCAGACATCTGTGTCC | GGGGTCTCTATGCCCAACAA |
| IFITM1 | CCAAGGTCCACCGTGATTAAC | ACCAGTTCAAGAAGAGGGTGTT |
| HLA-A | ACCCTCGTCCTGCTACTCTC | CTGTCTCCTCGTCCCAATACT |
| HLA-B | CAGTTCGTGAGGTTCGACAG | CAGCCGTACATGCTCTGGA |
| HLA-C | CCATGAGGTATTTGTGGACCG | TCTCGGACTCTCGTCGTCG |

**Supplementary Table1: Primers used in this study**
